# Similarities and differences in the nutritional composition of nuts and seeds in Serbia

**DOI:** 10.3389/fnut.2022.1003125

**Published:** 2022-09-16

**Authors:** Margarita Dodevska, Jelena Kukic Markovic, Ivana Sofrenic, Vele Tesevic, Milica Jankovic, Brizita Djordjevic, Nevena Dj. Ivanovic

**Affiliations:** ^1^Institute of Public Health of Serbia Dr. Milan Jovanovic Batut, Belgrade, Serbia; ^2^Faculty of Pharmacy, Department of Pharmacognosy, University of Belgrade, Belgrade, Serbia; ^3^Faculty of Chemistry, Department of Organic Chemistry, University of Belgrade, Belgrade, Serbia; ^4^The City Institute for Public Health, Belgrade, Serbia; ^5^Faculty of Pharmacy, Department of Bromatology, University of Belgrade, Belgrade, Serbia

**Keywords:** nuts, seeds, nutrients, essential amino acids, essential fatty acids, minerals, dietary fibers, phytosterols

## Abstract

Nuts and seeds are an indispensable part of the plant-based diet, which is becoming increasingly popular due to the evidence of their health benefits and contribution to sustainability and planetary health. Since the health effects of consuming nuts and seeds directly depend on their nutritional composition and consumed amount, it is essential to know the exact chemical composition of each nut and seed so that appropriate dietary interventions can be adequately planned. The present study aimed to examine the chemical composition of nuts and seeds obtained from the Serbian market and to highlight the similarities and differences in their nutritional composition. In twenty-four samples of nuts and seeds, the content of total lipids, including fatty acid profile, total proteins, including amino acid profile, total carbohydrates (sugars and fibers), phytosterols, and minerals were determined. Content of selected nutrients in grams (g) or milligrams (mg) of nuts and seeds was expressed in one portion (28 g) and as a percentage of contribution of Reference Intakes (% RI) and Dietary Reference Value (% DRV) for macronutrients and minerals, respectively. Some of the seed representatives appeared to be rich sources of essential omega-3 fatty acid, α-linolenic acid (ALA) (flax seed *vs*. walnut, 6.50 *vs*. 0.56 g per portion, respectively), dietary fibers (chia seed *vs.* raw almond, 10.6 *vs.* 3.4 g per portion, respectively), calcium (black sesame seed *vs*. almond roasted, 32.4% NRV *vs*. 8.1% NRV per portion, respectively), magnesium (hemp seed *vs.* Brazil nut, 38.3% NRV *vs*. 27.8% NRV per portion, respectively), and zinc (hemp seed *vs.* pine nut, 21.4% NRV *vs.* 17.6% NRV per portion, respectively). Our results highlighted the crucial role of seeds in the diet, especially as a better source of nutrients compared to nuts. Furthermore, it was seen that nuts and seeds are different and complementary in their composition. Thus, in order to meet the needs for certain nutrients for which nuts and seeds are used as dietary sources (essential fatty acids, minerals, dietary fibers), it would be beneficial to combine both nuts and seeds as a part of a healthy dietary pattern.

## Introduction

A growing body of evidence has demonstrated the health benefits of regular consumption of nuts and seeds which has aroused an increasing interest globally. Nuts and seeds are observed as essential components of healthy dietary patterns over the last decade (1). Moreover, nuts and seeds are an indispensable part of the plant-based diet, which is becoming increasingly popular due to the evidence of their health benefits and contribution to sustainability and planetary health (2). Also, cross-sectional studies and modeling approaches have demonstrated improved nutritional quality at the population level, especially when replacing other snack products with poor dietary quality (3).

As nuts and seeds have a similar nutrient profile and thus are expected to share similar mechanisms for their health effects. One of the strategies of specific national food-based dietary guidelines (FBDG) is to place nuts and seeds in the same group in order to increase their intake to recommended levels (2). In a significant number of FBDG, nuts and seeds are classified in the protein food group as alternatives to animal-based foods such as red meat, poultry, eggs, and seafood, although there are some FBDG in which nuts and seeds are classified in the group of oils/fats (4). For example, according to the Dietary Guidelines for Americans, 2020–2025, the intake of nuts and seeds should be at least 5 ounces per week. Moreover, one ounce of nuts and seeds (about 28 g) corresponds to two-ounce (56 g) of red meat, chicken, or fish and can meet one-third of the adult protein needs for men and two-fifths for women (5, 6).

In general, nuts and seeds are a high-energy, nutrient-dense plant group of foods, and a crucial component of the Mediterranean diet that has been shown to have a protective effect on cardiovascular disease development. In addition, the most extensive intervention study to date (PREDIMED study), which examined the health effects of nuts consumption for nearly 5 years and involved about 8,000 subjects with high cardiovascular risk, showed that a diet enriched with mixed nuts (walnuts, almonds, and hazelnuts), reduced systolic blood pressure, the incidence of metabolic syndrome and diabetes (7, 8). Furthermore, several studies have reported lower cardiovascular and all-cause mortality with every additional daily portion (28 g) of nuts (3). Therefore, several organizations, such as the World Health Organization and American Heart Association, officially encourage the consumption of nuts and seeds that are rich in protein and healthy fats and contain many other nutrients (9, 10). Nuts and seeds are important dietary sources of mono- and polyunsaturated fatty acids, including essential fatty acids, phytosterols, fibers, and polyphenols, with favorable mineral composition, especially with a low sodium content (7). Health effects are mostly related to the favorable composition of fatty acids and the suitable proportion of polyunsaturated and saturated fats (11). Nuts are also the first complete food that received a health statement. Namely, in 2003, the Food and Drug Administration (FDA) authorized a health statement referring to the beneficial effects of consuming about 43 g (around 1,5 recommended portion) of most nuts in reducing the risk of heart disease (12). Cashew nut is excluded from this health statement due to its high content of saturated fats. Further, in 2011, the European Food Safety Authority (EFSA) authorized a health statement linking the consumption of 30 g of walnuts per day as part of a balanced diet and improvements in endothelium-dependent vasodilation (13).

There is a difference in the chemical composition between the nuts and seeds group, as well as between the individual representatives of nuts and seeds. The difference is mainly in the content of proteins, fats, the ratio of mono and polyunsaturated fats, and the amount of specific vitamins and minerals and bioactive compounds. Furthermore, the chemical composition and nutritional value within the same representative of nuts and seeds may vary depending on plant variety, degree of ripening, agricultural practice, climatic conditions, including storage conditions and technological and thermal processes (14). Since the literature date indicate that the health effects of consuming nuts and seeds directly depend on their nutritional composition and the consumed amount, it is essential to know the chemical composition of nuts and seeds available on the market so that appropriate dietary intervention can be adequately planned. Namely, Serbia is a country with low to medium fish intake in the general population, also in this region, cardiovascular diseases are the leading cause of morbidity and mortality (15). Therefore, the adequate intake of seeds and nuts can contribute substantially to the fulfillment of needs for essential omega-3 fatty acids with proven cardioprotective effects and can also be a promising strategy for health promotion in this country (7).

Since many factors influence the nutrient composition of nuts and seeds and available literature on the chemical composition of certain types of nuts and seeds in samples from specific countries and regions is limited, this study aimed to obtain data on the nutritional value of nuts and seeds samples available in the Serbian market. In this work, chestnuts, which fall under the definition of nuts, were not considered due to their different nutritional composition, characterized by high starch content. In contrast, the work included peanuts, which are botanically classified as legumes, but due to their high-fat content and beneficial health effects, they are included in the nuts group. The second aim of this work was to estimate that upto which extent the intake of one recommended portion of nuts and seeds, proposed by USDA (1-ounce equivalent, around 28 g), is important to meet the needs for certain essential and non-essential nutrients and thus enable a comparison of nuts and seeds in terms of their place in a healthy diet of adults. Finally, this work intended to highlight the similarities and dissimilarities in the nutrient composition of nuts and seeds in order to estimate the significance of seeds next to nuts in current dietary recommendations.

## Materials and methods

### Samples collection

Twenty-four different types of nut and seed samples used in this study were purchased from several local markets in Belgrade in Serbia in 2021. Samples were collected as raw, boiled, or roasted. All samples were transferred to the laboratory in paper bags. Nuts and seeds were kept at + 4°C till analysis. For each sample, three replicate samples were analyzed. The samples were crushed with a mortar, and 100 g were taken to analyze lipids, including fatty acids and sterols, proteins, amino acids (AAs), and dietary fibers. The same weight was used to determine micro and macro elements after digestion.

### Reagents and solutions

All reagents were of analytical reagent grade unless otherwise stated. Ultra-pure water (resistivity > 18 MV cm) was obtained using a GenPure Water Purification System, Thermo Scientific, Thermoelectron LED, Langenselbold, Germany.

### Digestion procedure and elemental analysis

The mineral contents were analyzed using inductively coupled plasma optical emission spectrometry with an Agilent 5110 dual view, ICP-OES, and Inductively Coupled Plasma Mass Spectrometry (ICP-MS, 7900 × Agilent Technology (Waldbronn, Germany), after microwave-assisted acid digestion.

Milestone ETHOS UP, High-Performance Microwave Digestion System, Brondby, Denmark, was used for digestion. Samples weighing 0.3 g were digested with 9 ml of HNO_3_ (67–68%) and 1 ml of H_2_O_2_ (30%) in a microwave digestion system for 15 min, at 200°C, and finally diluted to 25 ml with deionized water. A blank digest was carried out in the same way. This process was conducted in triplicate. After the digestion process, the metal concentrations were determined using ICP-OES and ICP-MS.

External calibration was prepared from a multi-element stock solution of 50 mg/L: Cr, Zn; 10 mg/L: 100 mg/L: P; 200 mg/L: K; 100 mg/L: Mo, Na; 100 mg/L: Mg, Ca; 50 mg/L: Se; 20 mg/L: Fe, Cu; 20 mg/L: Mn (CPA chem mix 10 elements, Lot: 702961).

The limit of quantification (LOQ) is the concentration corresponding to ten times the standard deviation of 10 blanks. For quality control purposes, within each batch of samples analyzed, at least one procedural blank was included, in which no analytes were detected throughout the process. Recovery tests were performed, through the analysis of metal-spiked samples, with recovery efficiency for spiking sample analysis being at a level of 80–120%, following ISO 17025 norm criteria. The recovery was acceptable for all the elements determined.

### Determination of macronutrients

The nuts and seeds were analyzed for the percentage of moisture, proteins, fats, dietary fiber, and ash. Nutritional value was determined using the procedures suggested by the International Standards Association of Official Analytical Chemists [AOAC, (16)]. The moisture and crude ash content were estimated gravimetrically (AOAC Method 930.04 and AOAC Method 930.05, respectively), and the protein (N × 6.25) was determined by the Kjeldahl method (AOAC Method 977.02), and the crude fats by Soxhlet extraction with petroleum ether (boiling point, 40–60°C), (AOAC Method 930.09). Total carbohydrates were calculated as the residual difference after subtracting protein, crude ash, moisture, and crude fat content from 100.

### Determination of total fibers

The total fibers were determined by the enzymatic-gravimetric method (AOAC Method 985.29), described by Prosky et al. (17), using the complete dietary fibers assay kit K-TDFR (Megazyme Int., Wicklow, Ireland), The method requires phosphate buffer, pH 6.0 and the following enzymes: heat-stable α-amylase, protease, and amyloglucosidase. Heat stable α-amylase depolymerizes starch, protease depolymerizes and dissolves proteins, while amyloglucosidase converts starch into glucose. After the enzymatic treatment, the sample is corrected for possible mineral and nitrogen residue. Total dietary fibers were measured by gravimetric analysis.

### Determination of fatty acids

The fatty acid composition was determined using gas chromatography after converting fatty acid glycerides into the corresponding methyl esters, according to the ISO 12966-2 procedure (18). The analyses were performed using Agilent 7890a GC (Agilent 7890A, Santa Clara, CA, United States) with a flame ionization detector. Separations were made on a DB-5MS fused silica capillary column (60 m × 0.320 mm i.d.) with a film thickness of 0.25 μm. The fatty acid methyl esters (FAMEs) were identified by comparing the chromatogram’s retention times with a reference mixture of FAMEs (Supelco 37 component FAME mix).

### Determination of phytosterols

The sterol fraction was determined using gas chromatography with flame ionization detector (GC-FID) and gas chromatography with mass spectrometry (GC-MS) analysis of the complete unsaponifiable fraction according to the method described by Rabrenovic et al. (19). To analyze sterols and triterpenes, residual unsaponifiable fractions were treated with BSTFA (50 μl) and held at 60°C for 45 min to obtain volatile trimethylsilyl derivatives. The samples were analyzed within 6 h after derivatization. Gas chromatography analysis was performed on an Agilent 7890A GC (Santa Clara, CA, United States) equipped with 5975C (inert XL EI/CI) MSD and an FID detector connected by a capillary flow technology two-way splitter with make-up (250°C). An HP-5MS capillary column (Agilent, 30 m × 0.25 mm, 0.25 μm film thickness) was used. The compounds’ identification was based on comparing their retention indices (RI), retention time (Rt), and mass spectra from NIST/NBS 05, Wiley libraries 8th edition and NIST Chemistry WebBook.

### Determination of amino acids

Total AAs composition (Arginine, Serine, Valine, Methionine, Leucine, Alanine, Tyrosine, Threonine, Proline, Lysine, Isoleucine, Histidine, Phenylalanine, Glycine, Glutamate, Aspartate, and Cystine) were determined following an international standard EN ISO 13903, using GC-FID ion chromatography with the electrochemical detector, manufactured by Thermo, model ICS-5000, with a silver reference electrode (Ag/AgCl) and gold (Au) working electrode and chromatographic column AminoPac PA10 and pre-column AminoPac PA10 guard.

### Determination of sugars

Sucrose, D-glucose, and D-fructose were determined by a spectrophotometric method using the enzymatic assay kit R-biopharm (R-BIOPHARM AG, Darmstadt). The assay was performed according to the instruction manual of the kit producer. The D-glucose concentration was determined before and after hydrolysis of sucrose by β-fructosidase (invertase). The amount of NADH (nicotinamide adenine dinucleotide phosphate) formed in this reaction was proportional (directly correlated) to the D-glucose concentration and is measured by the increase in absorbance at 340 nm. The absorbance was measured by a Thermo Scientific, Evolution 201 spectrophotometer. The D-fructose content of the sample was determined subsequently to the determination of D-glucose after isomerization by phosphoglucose isomerase. The amount of NADH formed in this reaction was proportional to the amount of D-fructose concentration and was measured by the increase in absorbance at 340 nm. The sucrose content was calculated from the difference in D-glucose concentration before and after hydrolysis by β-fructosidase.

### Calculations

In this research study, the content of selected nutrients in grams (g) or milligrams (mg) of nuts and seeds was expressed in one portion. The portion of nuts and seeds was declared as 28 g, equivalent to one ounce, unit used in Dietary Guidelines for Americans to calculate the daily amount of different foodstuffs in the protein group (5). For calculation of contribution (in%) of macronutrients (fats, proteins, carbohydrates) and micronutrients (minerals) from one portion of nuts and seeds (28 g) in total recommended daily intake, Reference Intakes (RIs) (macronutrients) and Nutrient Reference Values (NRVs) (minerals) were used. RIs and NRVs are established by current EU regulation (Regulation (EU) No 1169/2011 of the European Parliament and of the Council of 25 October 2011 on the provision of food information to consumers) and are used for the labeling of foodstuffs, representing needs of an average adult with energy needs of 8,400 kJ/2,000 kcal (20). Content of AAs in referent protein was used to calculate the contribution (in%) of essential amino acids from one portion of nuts and seeds, based on a Report of a Joint WHO/FAO/UNU Expert Consultation (21). Limiting essential AA was defined as an essential amino acid (EAA) with the lowest content in nuts and seeds compared with its content in the referent protein.

### Statistical analysis and principal component analysis

The statistical data analysis was done using SPSS 20.0 (IBM Corp., Armonk, NY, United States) and Microsoft Excel 2013 (Microsoft, Redmond, WA, United States). Fundamental statistical analysis included arithmetic mean and standard deviation. Principal component analysis (PCA) was employed to observe correlations between analyzed parameters (elements) and examine similarities between the individual nut and seed samples. The biplot construction has achieved the visualization.

## Results

### Nuts and seeds as a source of lipids, essential fatty acids, and phytosterols

The content of total fat, saturated fatty acids (SFAs), oleic (OA), linoleic (LA), and α-linolenic acid (ALA) in nuts and seeds are presented in Table 1. All results were expressed in grams (g) per portion (28 g), with the content of total fats and SFAs also expressed as a percentage of contribution of Reference Intake (% RI). Total fats and individual fatty acids in g/100 g are given in Supplementary Table 1.

**TABLE 1:** Total fat, saturated fatty acids, oleic acid, linoleic acid, and α-linolenic acid content and contribution to RI (%) of fats and SFA from one portion (28 g) of nuts and seeds.

Sample	Total fat (g)	% RI fat	SFAs* (g)	% RI SFA	OA (g)	LA (g)	ALA (g)	UFAs/SFAs**
Peanut raw	14	20	2	10	7	4	< 0.05	6.2
Peanuts roasted	15	21	2	12	7	4	< 0.05	5.0
Peanuts boiled	14	20	2	10	7	4	< 0.05	6.3
Almond raw	14	20	1	5	9	3	< 0.05	12.4
Almond roasted	15	22	1	6	10	4	< 0.05	12.1
Almond boiled	14	20	1	5	9	3	< 0.05	12.0
Hazelnuts raw	17	24	1	6	13	2	< 0.05	12.5
Hazelnuts roasted	17	25	1	6	13	2	< 0.05	12.9
Walnut	17	25	1	5	4	9	0.56	17.5
Brazil nut	19	27	4	21	7	6	< 0.05	3.4
Cashew nut raw	12	18	2	11	7	2	< 0.05	4.6
Cashew nut roasted	13	19	2	12	7	2	< 0.05	4.6
Pecan nut	20	29	2	9	11	6	0.28	10.6
Pine nuts	19	27	1	7	5	9	< 0.05	13.0
Pistachios roasted	12	18	2	8	6	4	0.07	7.2
Sunflower seed raw	14	21	1	10	5	6	< 0.05	10.4
Sunflower seed roasted	14	18	2	12	2	10	< 0.05	6.2
Pumpkin seed raw	13	17	2	11	4	6	0.05	4.3
Pumpkin seed roasted	12	12	2	4	4	5	0.05	4.3
Chia seed	9	17	1	5	1	2	4.91	8.6
Flax seed	12	20	1	6	2	2	6.50	10.4
Hemp seed	14	20	1	10	1	8	2.43	9.6
Sesame seed	14	21	2	6	5	6	0.11	6.1
Black sesame seed	18	26	3	13	7	8	0.14	6.2

RI, Reference intakes of an average adult (8,400 kJ/2,000 kcal) established for energy and selected nutrients other than vitamins and minerals for adults by the European Parliament (RI for total fat = 70 g; RI for saturated fat = 20 g) (20); SFAs, saturated fatty acids; OA, oleic acid; LA, linoleic acid; ALA, α-linolenic acid; UFA, unsaturated fatty acid. *Expressed as the sum of myristic, palmitic, stearic, and arachidic acid. **The ratio of the sum of unsaturated fatty acids to the sum of saturated fatty acids.

In portion (28 g) contribution of nuts to total fats intake was in the range of 18% RI (pistachio and cashew nut) to 29% RI (pecan nut), while in seeds, these values ranged from 12% RI (chia seed) up to 26% RI (black sesame).

A significant difference in SFA content between individual nuts and seeds was observed, with the highest content in brazil nuts contributing almost one-fifth of RI for SFA. In peanuts and cashew nuts, this contribution was two times lower (10–12% and 10–11% RI, respectively) and almost four times lower in portions of hazelnuts, almonds, and walnut (5–6% RI). SFA content was lower than nuts and ranged from 4% RI (chia seed) to 13% RI (black sesame).

The fatty acid profile of nuts is dominantly represented by monounsaturated fatty acids (MUFA), namely oleic acid, with walnut being the only exception: it was rich in polyunsaturated fatty acids (PUFA). On the other hand, the fatty acid profile of seeds was characterized by PUFA, mainly linoleic acid (LA), except for chia and flaxseed, in which the main PUFA was alpha-linoleic acid (ALA) (Supplementary Table 1). Along with flaxseed (23.2 g/100 g) and chia seed (17.5 g/100 g), hemp seed also could be considered a significant source of ALA (8.7 g/100 g) among all investigated samples. Walnuts were the richest source of ALA among the investigated nuts (2 g/100 g), still having multiple lower ALA content than the seeds mentioned earlier. Pecan nuts could be considered a significant source of ALA, fulfilling up to 90% of the recommended intake for ALA (Supplementary Table 1).

Sterol content in the investigated samples, expressed in mg per portion, is presented in Table 2, while values in g/100 g are shown in Supplementary Table 2. Total sterol content in one recommended portion of nut samples ranged from 22.2 mg (roasted cashew nut) up to 77.6 mg (pine nuts) and was higher in seeds, ranging from 43.4 mg (roasted pumpkin seed) to 141.6 mg (black sesame seeds).

**TABLE 2:** Phytosterol content (in mg) in one portion (28 g) of nuts and seeds.

Sample	Squalene	Campesterol	Stigmasterol	β -Sitosterol	Isofucosterol	Cycloartenol	Δ 7-Avenasterol	Citrostadienol	Total sterols
Peanut raw	3.7	4.9	4.2	19.8	3.2	0.5	0.2	0.2	36.6
Peanuts roasted	2.4	4.4	3.9	18.4	2.7	0.4	0.1	0.2	32.5
Peanuts boiled	3.5	4.0	3.3	16.2	2.4	0.2	0.1	0.2	29.8
Almond raw	0.8	1.4	< 0.1	38.0	5.0	0.5	0.7	0.4	46.8
Almond roasted	1.0	1.3	0.3	40.4	4.7	1.0	0.9	0.8	50.4
Almond boiled	0.9	0.9	< 0.1	31.5	3.2	0.9	0.8	0.7	38.7
Hazelnuts raw	4.4	1.6	0.4	29.4	0.8	< 0.1	0.3	1.0	37.9
Hazelnuts roasted	6.0	1.6	5.3	23.9	0.6	< 0.1	0.1	0.6	38.2
Walnut	< 0.1	1.2	< 0.1	23.6	2.1	2.5	< 0.1	0.8	30.3
Brazil nut	31.2	< 0.1	1.9	3.3	< 0.1	0.1	0.1	0.7	37.2
Cashew nut raw	1.2	1.7	0.1	21.6	2.4	1.0	< 0.1	0.3	28.3
Cashew nut roasted	0.9	1.5	< 0.1	16.8	2.0	0.8	< 0.1	0.2	22.2
Pecan nut	2.0	1.1	0.7	28.0	3.4	0.7	0.1	1.4	37.4
Pine nuts	< 0.1	4.6	< 0.1	38.8	28.6	1.1	0.8	3.7	77.6
Pistachios roasted	1.6	3.7	0.7	57.7	5.0	< 0.1	0.3	1.7	70.7
Sunflower seed raw	2.8	4.9	4.9	39.2	1.4	10.9	2.6	8.2	74.9
Sunflower seed roasted	2.2	5.2	4.3	39.4	2.7	7.4	2.4	5.4	69.0
Pumpkin seed raw	9.2	< 0.1	<0.1	29.8	< 0.1	<0.1	7.5	< 0.1	46.5
Pumpkin seed roasted	8.7	< 0.1	<0.1	27.6	< 0.1	<0.1	7.1	< 0.1	43.4
Chia seed	0.1	< 0.1	<0.1	37.5	< 0.1	<0.1	0.4	2.1	40.1
Flax seed	0.2	12.1	5.8	26.1	5.7	11.5	< 0.1	0.8	62.4
Hemp seed	0.1	7.8	1.1	37.8	3.2	1.8	0.3	0.9	53.0
Sesame seed	0.3	21.2	8.0	85.3	15.7	1.4	1.6	3.6	137.1
Black sesame seed	0.3	23.6	9.6	80.6	22.2	1.3	1.3	2.8	141.6

The main sterol in all samples was β-sitosterol, with campesterol, stigmasterol, and isofukosterol as dominant sterols in a few other samples, but with significantly lower content.

### Nuts and seeds as a source of proteins and essential amino acids

Protein content expressed as grams (g) and % of RI for proteins per portion, as well as the content of essential amino acids (EAAs) present in referent protein, expressed as % of established needs for an average adult are presented in Table 3.

**TABLE 3:** Protein content, percentage of reference intake (RI) for proteins and contribution to essential amino acids requirements (EAAR) from one portion (28 g) of nuts and seeds.

Sample	Protein (g)	% RI* protein	% EAAR** Lys	% EAAR Thr	% EAAR Val	% EAAR Ile	% EAAR Leu	% EAAR Met	% EAAR His	% EAAR Phe
Peanut raw	8.2	16.4	14.4	23.2	16.5	329.2	20.2	10.7	28.4	21.8
Peanuts roasted	8.0	16.0	8.7	20.0	10.2	203.1	22.1	20.0	21.2	16.6
Peanuts boiled	8.3	16.6	13.9	26.1	16.2	323.1	17.4	5.1	32.4	23.4
Almond raw	6.8	13.6	10.1	14.7	13.4	267.7	14.2	3.7	23.2	18.9
Almond roasted	7.3	14.5	9.3	14.1	12.6	252.3	15.3	4.0	27.2	20.6
Almond boiled	7.5	15.0	10.3	24.3	14.9	298.5	24.3	8.8	26.4	20.5
Hazelnuts raw	4.7	9.5	7.1	13.6	11.2	224.6	11.4	4.3	20.0	11.8
Hazelnuts roasted	5.1	10.1	7.5	16.3	11.5	230.8	12.2	5.6	22.0	14.6
Walnut	5.3	10.5	8.3	16.5	11.4	227.7	12.2	5.3	22.4	13.6
Brazil nut	4.6	9.1	5.5	14.1	7.2	144.6	11.7	18.4	14.8	7.4
Cashew nut raw	6.0	12.0	14.7	19.7	17.1	341.5	14.9	7.2	24.0	15.5
Cashew nut roasted	6.2	12.3	14.1	25.6	14.9	298.5	17.2	11.7	21.6	14.1
Pecan nut	3.3	6.5	4.7	7.2	5.5	110.8	6.8	3.7	13.2	7.0
Pine nuts	4.9	9.8	9.2	14.9	9.8	196.9	11.4	8.0	17.6	9.0
Pistachios roasted	6.4	12.9	15.1	22.9	15.4	307.7	18.1	12.0	20.0	14.7
Sunflower seed raw	5.9	11.8	12.5	22.9	15.2	304.6	13.6	11.5	32.0	15.7
Sunflower seed roasted	5.7	11.5	8.9	19.2	12.9	258.5	14.1	12.0	26.8	13.3
Pumpkin seed raw	9.3	18.6	15.3	28.3	21.5	430.8	32.8	23.7	37.6	24.2
Pumpkin seed roasted	10.0	20.1	19.6	32.8	29.7	593.8	30.6	20.3	44.4	33.3
Chia seed	5.7	11.5	15.3	28.3	11.7	233.8	10.9	13.3	26.8	15.0
Flax seed	6.6	13.2	12.9	23.2	15.4	307.7	13.8	11.7	22.4	14.6
Hemp seed	9.6	19.2	21.9	29.9	29.1	581.5	20.1	26.9	42.4	32.5
Sesame seed	7.5	14.9	12.0	32.5	19.7	393.8	20.4	19.2	39.2	21.6
Black sesame seed	5.8	11.6	11.2	20.8	15.7	313.8	11.6	17.9	24.4	19.0

*RIs – Reference intakes of an average adult (8 400 kJ/2 000 kcal) established for energy and selected nutrients other than vitamins and minerals for adults by the European Parliament (RI for protein = 50 g). **EAAR – essential amino acid requirements according to Report of a Joint WHO/FAO/UNU Expert Consultation (21); Lys, L-lysine; Thr, L-threonine, Val, L-valine; Leu, L-leucine; Ile, L-isoleucine; Met, L-methionine; His, L-histidine; Phe L-phenylalanine.

The obtained data showed that seeds represent a more significant source of proteins than nuts, with roasted pumpkin seed and hemp seed having the highest protein content (10.0 and 9.6 g/portion, respectively), obtaining up to 20.1 and 19.2% RI, respectively. Among nuts, the most significant sources of proteins were peanuts, almond, and cashew nut samples, with slight differences between raw and roasted ones, providing 12.0–16.6% RI. As for EAAs, it could be observed that in most nuts, methionine was the first limiting AA (present in the lowest% compared to a referent AA), while lysine was the second. The only exception was brazil nuts with lysine as the first and valine as the second limiting AA. Within seeds, the results were the opposite: lysine was always the first limiting AA, except for chia and black sesame seed, in which the first and second limiting AA were leucine and methionine, respectively. Also, differences in limiting AA between raw and roasted samples of peanuts and pumpkin seeds could be attributed to different sources of samples.

Content of essential and non-essential amino acids (EAAs and NEAAs, respectively) is given in Supplementary Tables 3A,B, while total protein content in samples (g/100 g) along with complete amino acid profile in samples (g/100 g) is presented in Supplementary Table 4. EAAs content in total AAs (Supplementary Table 4) in nuts ranged from 25.27% (roasted almonds) to 36.94% (roasted cashew nuts), while in seeds, it was somewhat lower, ranging from 31.95% (flax seeds) up to 39.63% (raw pumpkin seeds).

### Nuts and seeds as a source of minerals

The content of macro- and micro-essential minerals is presented in Table 4. expressed as the contribution of their intake from one portion (28 g) of nuts and seeds, in% of NRV established for minerals (20).

**TABLE 4:** Contribution (in%) of macro- and microessential minerals intake from one portion (28 g) of nuts and seeds to nutrient reference values (NRV).

Sample	Na% NRV	K% NRV	Ca% NRV	Mg% NRV	P% NRV	Fe% NRV	Cu% NRV	Zn% NRV	Cr% NRV	Mo % NRV	Mn% NRV
Peanut raw	0.1	4.9	2.3	12.7	16.9	7.1	29.1	9.7	30.7	49.3	19.2
Peanuts roasted	0.1	5.3	2.5	13.8	18.3	7.6	27.8	10.3	26.2	108.1	20.2
Peanuts boiled	–	5.1	1.2	11.6	15.3	3.8	9.9	8.5	23.9	99.1	19.3
Almond raw	< 0.1	5.5	7.0	19.8	20.8	21.4	30.0	8.8	151.9	13.8	28.8
Almond roasted	< 0.1	5.7	8.1	19.9	21.3	15.8	26.9	9.0	132.3	25.9	29.0
Almond boiled	< 0.1	5.6	7.9	19.9	21.1	17.0	28.3	9.0	139.3	16.0	28.8
Hazelnuts raw	–	5.3	4.9	11.0	13.6	9.2	37.2	6.5	119.0	–	89.9
Hazelnuts roasted	–	5.6	4.9	11.3	14.5	5.7	38.6	6.6	–	–	100.8
Walnut	–	5.6	3.4	13.9	21.5	7.0	44.2	10.2	70.7	–	42.8
Brazil nut	–	5.3	5.6	27.8	29.0	5.2	52.6	11.7	–	–	18.5
Cashew nut raw	0.1	5.3	1.3	17.7	21.0	10.3	55.4	14.4	–	–	25.2
Cashew nut roasted	5.4	5.5	1.8	18.7	22.1	11.5	61.9	16.1	–	–	28.4
Pecan nut	–	3.2	1.9	7.8	11.3	5.2	21.0	10.5	–	–	49.7
Pine nuts	–	4.8	0.3	14.5	19.8	8.4	25.6	17.6	–	–	102.2
Pistachios roasted	1.0	6.2	3.5	7.3	18.4	5.4	13.0	6.0	21.7	–	11.0
Sunflower seed raw	–	5.4	3.5	21.0	28.4	8.1	48.7	14.8	–	21.0	29.8
Sunflower seed roasted	3.1	3.8	1.9	13.7	18.2	4.9	28.0	9.7	–	14.3	21.1
Pumpkin seed raw	0.1	5.7	2.0	31.8	37.2	14.0	25.3	9.3	18.2	81.8	41.0
Pumpkin seed roasted	4.0	6.1	2.1	32.4	39.9	14.8	28.6	12.7	21.0	85.1	44.9
Chia seed	–	6.3	20.4	25.5	36.9	12.4	39.8	14.7	22.4	32.4	57.7
Flax seed	0.9	6.1	5.5	22.7	24.8	11.0	29.7	14.1	18.9	–	36.7
Hemp seed	< 0.1	6.1	4.9	38.3	38.7	30.4	35.3	21.4	33.6	41.6	84.3
Sesame seed	0.7	4.1	2.2	21.9	25.9	11.3	43.1	15.5	–	45.5	18.2
Black sesame seed	–	3.9	32.4	18.6	21.9	11.1	37.0	11.5	–	142.8	23.9

Generally, analyzed nuts and seeds had low sodium content, except for roasted samples in which salt was added during the roasting process (Table 4). Though analyzed samples contained significant amounts of potassium ranging from 2,708 mg/kg (roasted sunflower seeds) to 4,478 mg/kg (chia seeds) (Supplementary Table 5), consuming the recommended 28 g portion of nuts and seeds will cover just 3.8%–6.3% NRV for potassium. As for magnesium, among nuts, brazil nuts, almonds, cashew nuts roasted, and walnuts could be considered important sources of this mineral since one recommended portion will contribute to NRV with 27.8, 19.9, 18.7, and 13.9%, respectively. Among seeds, the most valuable magnesium sources were hemp seeds and pumpkin seeds, contributing 38.3, 32.4, and 31.8% NRV with one portion, respectively. One portion of nuts and seeds contributes 0.3–32.4% NRV for calcium, with almonds having the highest value among nuts (8% NRV), while within seeds, chia (5,836 mg/kg, 20.4% NRV per portion) and black sesame (9,247 mg/kg, 34.7% NRV per portion) could be considered as the most valuable sources of this mineral. As for iron, the most significant sources among nuts were raw and roasted almonds (21.4 and 17.0% NRV per portion, respectively), and among seeds, these are pumpkin seeds (*ca*. 15% NRV) and hemp seed (30.4% NRV). Results presented in Table 4 indicate seeds as a more significant nutritional source of zinc than nuts. One recommended portion of seeds, depending on the source, contributes 6.0% NRV (pistachios) to 17.6% NRV (pine nuts) for zinc, while this value is significantly higher in seeds, ranging from 9.3% NRV (raw pumpkin seeds) up to 21.4% NRV (hemp seeds). All analyzed nuts and seeds samples could be considered a valuable source of copper and manganese (except almonds boiled and pistachios).

### Nuts and seeds as a source of dietary fiber

Contents of total carbohydrates, fibers, and sugars in one recommended portion are presented in Table 5 and Supplementary Table 6.

**TABLE 5:** Total carbohydrates, fibers, sugars, glucose, fructose, and sucrose content in one recommended portion (28 g) of nuts and seeds.

Sample	Total carbohydrates (g)	Total fibers (g)	Total sugars* (g)	Sucrose (g)	Glucose (g)	Fructose (g)	Carbohydrates/fibers**	Fibers/sugars***
Peanut raw	3.9	2.4	1.1	1.1	< 0.1	<0.1	1.7	2.1
Peanuts roasted	4.6	2.4	1.2	1.1	< 0.1	<0.1	1.9	2.0
Peanuts boiled	4.8	2.4	1.1	1.1	< 0.1	<0.1	2.0	2.1
Almond raw	5.7	3.4	1.1	1.0	< 0.1	<0.1	1.7	3.1
Almond roasted	4.3	2.9	1.1	1.2	< 0.1	<0.1	1.5	2.6
Almond boiled	5.1	2.9	1.4	1.3	< 0.1	<0.1	1.7	2.1
Hazelnuts raw	4.5	2.7	1.2	1.2	< 0.1	<0.1	1.7	2.3
Hazelnuts roasted	4.5	2.6	1.4	1.3	< 0.1	<0.1	1.7	1.9
Walnut	2.7	2.0	0.4	0.4	< 0.1	<0.1	1.4	5.5
Brazil nut	3.4	2.1	0.6	0.7	< 0.1	<0.1	1.6	3.3
Cashew nut raw	7.9	0.9	1.7	1.6	< 0.1	<0.1	8.6	0.6
Cashew nut roasted	7.3	0.9	1.4	1.3	< 0.1	<0.1	8.2	0.6
Pecan nut	3.9	2.7	1.1	1.1	< 0.1	<0.1	1.5	2.4
Pine nuts	3.0	1.0	1.0	1.0	< 0.1	<0.1	2.9	1.0
Pistachios roasted	7.7	2.9	2.1	2.0	0.1	< 0.1	2.7	1.4
Sunflower seed raw	5.9	2.4	0.7	0.7	< 0.1	<0.1	2.5	3.3
Sunflower seed roasted	7.1	3.0	0.9	0.8	< 0.1	<0.1	2.4	3.4
Pumpkin seed raw	3.4	1.1	0.5	0.3	0.1	0.1	3.1	2.1
Pumpkin seed roasted	3.6	1.1	0.6	0.4	0.1	0.1	3.3	1.9
Chia seed	11.6	10.6	< 0.1	<0.1	< 0.1	<0.1	1.1	–
Flax seed	7.6	7.1	0.4	0.3	0.1	< 0.1	1.1	15.8
Hemp seed	1.9	1.1	0.4	0.2	0.1	0.1	1.7	2.7
Sesame seed	4.9	3.3	0.1	0.1	< 0.1	<0.1	1.5	39.3
Black sesame seed	2.9	2.8	0.1	0.0	< 0.1	<0.1	1.1	50.0

*Expressed as the sum of glucose, fructose, and sucrose. **The ratio of carbohydrates to fibers. ***The ratio of fibers to sugars.

Total fiber content indicates that nuts and seeds are their important source, with raw almonds being the richest source among nuts (12.2 g/100 g) and chia seeds among seeds and all samples (37.7 g/100 g). When fibers content is expressed per one portion, their contribution to daily fibers intake is also evident: in nuts, total fibers content in one portion ranges from 0.9 g (cashew nuts) to 3.4 g (raw almonds), while in seeds, it is varied from 1.1 g (pumpkin and hemp seeds) to 10.6 g (chia seed).

As for total carbohydrates and sugars, observed differences among samples are insignificant compared to recommended portions.

From the carbohydrates/fibers ratio (Table 5), it is evident that investigated samples contain somewhat more carbohydrates than fibers (*ca*. 1.5–2.5 times), with few exceptions (cashew nut raw (8.6) and roasted (8.2) and pumpkin seed raw (3.1) and roasted (3.3). Fibers/total sugar ratio was much higher for nuts and seeds, especially in black sesame seeds (50.0) and sesame seeds (39.3), while cashew nuts were the only ones showing the opposite trend (0.6).

### Principal component analysis

Principal component analysis was applied to integrate results of chemical parameters, discover the possible correlations among measured parameters, and classify the parameters in a factor plane. Also, PCA is widely used to find the relationships between variables and sample types. PCA is a factor model in which the factors are based on summarizing the total variance. The first two factors should correspond to a high% of the variance to ensure that the maps based on the first two factors are a good quality projection of the initial multi-dimensional table.

The analyzed nutrients of nuts and seeds were used to generate the PCA model. Statistical parameters were calculated for nuts and seeds separately to recognize and explain differences influenced by selected parameters.

**FIGURE 1:**
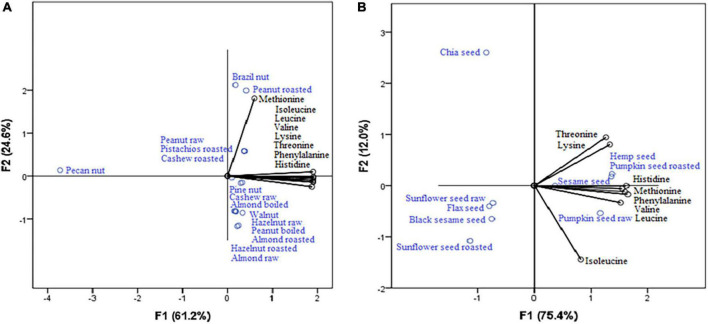
Nuts **(A)** and seeds **(B)** as a source of essential amino acids.

According to PCA for nuts (Figure 1A), the highest content of histidine, valine, phenylalanine, lysine, threonine, and leucine (which show strong positive correlation with the first axis: 0.986, 0.969, 0.951, 0.823, 0.772, and 0.655, respectively) were observed in raw peanut, roasted pistachios and cashew nut roasted (it can also be seen from their position), while brazil nut is the most significant source of methionine (strong positive correlation with the second axis: 1.007). Within seeds (Figure 1B), the position of hemp seed, pumpkin seed roasted, and sesame seed indicates that they are the highest sources of lysine, threonine, phenylalanine, valine, histidine, and methionine (strong positive correlation with the first axis: 1.038, 0.939, 0.904, 0.841, 0.797, and 0.643, respectively). Isoleucine and leucine were connected to the second axis, revealing pumpkin seed raw as the richest source of these EAAs. Pecan nuts (Figure 1A) and chia seeds (Figure 1B) contain the least amount of amino acids, and this was confirmed by the graphs which show that the amino acids are the most distant from them.

**FIGURE 2:**
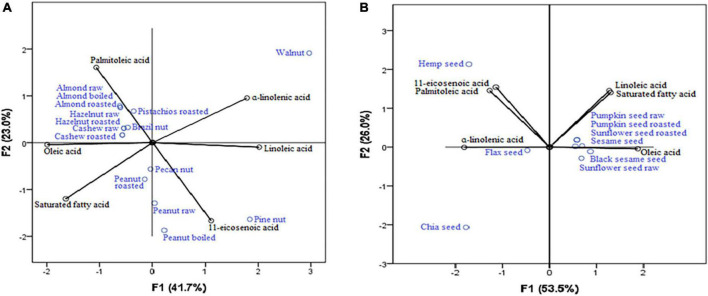
Nuts **(A)** and seeds **(B)** as a source of fatty acids.

Results of PCA (Figure 2A) revealed the highest concentrations of 11-eicosenoic acid (strong positive correlation with the first axis: 0.864) were present in pine nut, pecan nut, peanut raw, and peanut boiled, while all nuts could be considered rich sources of oleic acid (strong negative correlation with the first axis: −0.747), with exceptions of pine nuts and walnuts. The most important sources of α-linolenic acid (strong negative correlation with the first axis: −0.936) were flax seeds and chia seeds (Figure 2B).

**FIGURE 3:**
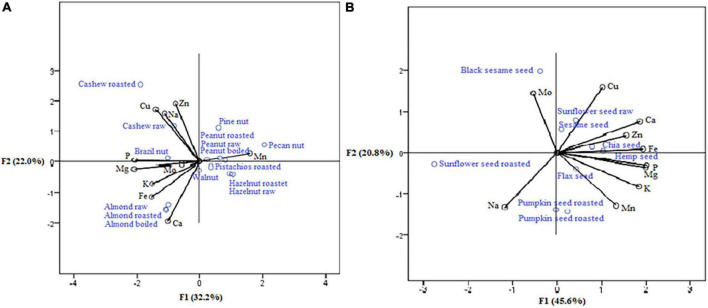
Nuts **(A)** and seeds **(B)** as a source of minerals.

The positions of nuts, such as almond raw, almond roasted, and almond boiled (Figure 3A) were seen to be the essential sources of calcium and iron (strong correlation with the first axis: 0.916 and 0.730, respectively), while at the same time, cashew nut raw and cashew nut roasted were the richest in zinc and cooper (strong correlation with the second axis: 0.915 and 0.763, respectively). Within seeds samples (Figure 3B), the highest concentrations of manganese, potassium, magnesium, phosphorus, iron, and zinc (strong positive correlation mainly with the first axis: 0.932, 0.827, 0.967, 0.900, 0.883, and 0.618, respectively) were observed in pumpkin seed raw, pumpkin seed roasted, flax seed, hemp seed, and chia seed (according to their position). Chia seed was the richest source of calcium (positive correlation with the second axis: 0.678).

**FIGURE 4:**
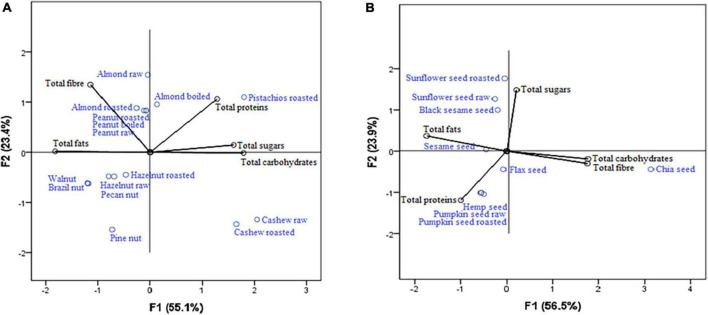
Nuts **(A)** and seeds **(B)** as a source of macronutrients.

Principal component analysis considering macronutrients in nuts (Figure 4A), highlighted cashew nut raw and cashew nut roasted as important sources of carbohydrates and total sugars (strong correlation with the first axis: 0.969 and 0.854, respectively), while in seeds (Figure 4B), chia seed is recognized as the most important source of carbohydrates and fibers within (strong correlation with the first axis: 0.727 and 0.667, respectively).

## Discussion

To give the actual frame of nuts and seeds consumed in the population and to compare their nutrient profile, in this study, commercial samples of nuts and seeds are analyzed and discussed regarding their position in the diet. Reference Intakes (RIs) and Nutrient Reference Values (NRVs) that were used for the calculation of the contribution of macronutrients and minerals to daily intake, respectively, were established according to European Union regulation on the provision of food information to consumers and are considered as guidelines for consumers on approximate amounts of macronutrients, micronutrients, and energy required by an average adult person for a healthy diet (20). The expression of macronutrient content in one portion of nuts and seeds as a percent of reference intakes (% RI) for macronutrients and as a percent of nutrient reference values (% NRV) for minerals could be obtained more precise insight into the significance of certain nuts and seeds in nutrition. Namely, literature data point out that the beneficial effects of consuming nuts are dose-dependent and usually are expressed as benefits that could be achieved by consuming an extra daily portion of nuts or by increasing the frequency of their consumption. Still, there are suggestions that this dose-dependency is probably curve-shaped and that at some point, such increased consumption could result in increased intake of fats, and consequently in increased body weight, since nuts are considered high-energy-dense foods, primarily because of fats high content (2).

Furthermore, though certain FBDG-recommend nuts and seeds as a part of a healthy dietary pattern, the health effects of seeds are not as comprehensively investigated as nuts. Namely, investigations concerning the health effects of seeds alone or in combination with nuts are substantially scarce. These investigations do not refer to seeds’ oil since oils are classified as a separate group of food in the majority of guidelines and represent just one of many nutrients in seeds, which all together contribute to the beneficial effects of seeds in diet (2). Results of the meta-analysis, which included 28 randomized studies (RCTs), showed that dietary intervention with flaxseed, but not flaxseed oil, decreases total and LDL cholesterol, while another meta-analysis of 11 RCTs also showed a more significant effect of flaxseed compared to flaxseed oil on lowering of systolic and diastolic blood pressure (2, 22, 23). Moreover, because of the limited ability of some dietary assessment methods to evaluate the intake of seeds as a part of commonly used food (muesli, granola, cereals, and different types of bread) or lack of information on the nutritional compositions of seeds in nutrient databases, intake of seeds in the nutritional analysis is sometimes neglected. Thus, there are certain doubts that the presence of seeds in food partially influences health effects attributed to nuts, so it is necessary to revise the current opinion on nuts to include seeds, which would contribute significantly to planning future investigations and new dietary guidelines (2). Also, as a consequence of an insufficient number of studies concerning seeds exclusively, there are no health statements referring to seeds.

Results of our analysis of fats and fatty acid content (Table 1) are in agreement with data from the USDA National Nutrient Database (24) except for walnut with twofold lower saturated fatty acid (SFA) content (3.4 g/100 g *vs* 6.1 g/100 g) (Supplementary Table 1). Based on the presented data, it is clear that for some representatives of nuts and seeds, above all for those with low fat and SFA content such as walnut, almonds, hazelnuts, chia, and sunflower seeds, an additional daily portion could be recommended. For some investigated nuts, such as brazil nuts, cashew nuts, and peanuts, an additional portion could contribute to excessive intake of SFAs, especially in combination with other foodstuffs that are also a significant source of SFAs (meat, dairy products) and are necessary for a well-balanced and healthy diet.

Omega-3 fatty acids possess a wide range of potential health effects, including regulation of inflammation and antioxidant signaling pathways, thus providing cardioprotective effects (25). Long-chained omega-3 fatty acids (FAs), such as eicosapentaenoic acid (EPA) and docosahexaenoic acid (DHA), which could be, to a certain extent, biotransformed from ALA, found only in plant-based foods, are vital for growth, cognition, and immune system function (26). Fish are the most important source of these fatty acids, but in populations that have an insufficient fish intake in their diet, there is a risk of low dietary intake of these FAs. That is why including alternative nutritional sources of omega-3 FAs, such as various seeds and walnuts in the first place, is of great importance. A study comparing the effects of consuming 30 g of raw sunflower seed and the same amount of almonds in postmenopausal women with type 2 diabetes showed that both interventions lowered total and LDL cholesterol for 3 weeks. However, intervention including sunflower seeds showed more pronounced effects on decreasing triglycerides, HDL, apoA-1, and B-100, which could be explained by higher polyunsaturated FA (PUFA) content in sunflower seeds (27). According to EU regulations on the application of nutrition and health claims, all foodstuffs containing more than 0.3 g ALA/100 g and 0.6 g ALA/100 g could be labeled as “sources” and “rich sources” of omega-3 fatty acids, respectively (28). This is in line with these definitions and based on the results of the present investigation shown in Supplementary Table 1. Regarding the content of ALA, it could be concluded that sesame and black sesame seed should be considered sources of omega-3 FA, while walnut, chia, flax, and hemp seeds could be labeled as rich sources of omega-3 FA.

Our results also highlight that combining nuts rich primarily in monounsaturated fatty acids (MUFA) with seeds that are sources of PUFAs, mainly essential fatty acids LA and ALA, could increase the proportion of PUFA and significantly fulfill recommended intake of essential FA. Moreover, combining nuts and seeds could provide a satisfactory effect on blood lipid profile because of the different mechanisms of hypocholesterolemic action, most likely due to the different profiles of MUFAs and PUFAs between them (2). The total unsaturated fatty acids/saturated fatty acids (UFA/SFA) ratio presented in Table 1 showed significant differences between investigated nuts and seeds: a higher ratio was generally observed in nuts and they ranged from 3.4 (brazil nuts) to 17.5 (walnuts). In contrast, in seeds, it ranged from 4.3 (pumpkin seed) up to 10.4 (raw sunflower seeds and flax seeds). Results obtained in our work were only partly in agreement with the results of other authors. For example, UFA/SFA ratios in our experiments for hazelnuts were 1.5-fold higher (12.5 *vs* 8.47), while for pumpkin seed, it was twofold lower (4.3 *vs* 8.6) compared with the study of Kirbaslar et al. (29). The discrepancy with literature data can be explained by the fact that the composition of nuts and seeds is determined by a large number of factors, including plant variety, degree of ripening, agricultural practice, climatic conditions as well as storage conditions (14). This UFA/SFA ratio was also used previously in some investigations (29, 30), permitting a better understanding of the fatty acid profile of nuts and seeds and also being important as a predictive factor for shelf life (29). Based on data obtained in our research, a lower UFA/SFA ratio for seeds implicates their longer shelf life under the same conditions as nuts. It was also interesting to observe significant differences between this ratio’s values concerning raw and roasted sunflower seeds, which is probably influenced by different origins of raw materials for these commercial samples.

As for phytosterol content in analyzed nuts and seeds, our results are, in some cases, considerably different compared to literature data that could be explained by other analytical procedures, sample preparation, and above all, by the different origins of the samples tested. Nevertheless, β-sitosterol was also reported as the main phytosterol in nuts and seeds by many other authors (31, 32). In samples of nuts and seeds usually consumed in the United States, Phillips et al. (32) reported the highest amount of phytosterols in sesame seeds (400–413 mg/100 g) and the lowest in brazil nuts (95 mg/100 g). In our investigation, sesame and black sesame seed were also the most important source of phytosterols (489.6–505.5 mg/100 g, Supplementary Table 2), with the lowest phytosterol content in roasted cashew nut, boiled peanut, and walnut (79.3, 106.5, and 108.4 mg/100 g, respectively). As for phytosterols in brazil nuts, a sample from our investigation contained 132.9 mg/100 g, which is higher than in the respective research from the United States. Phytosterols are well-known for their cholesterol-lowering effects through competitive mechanisms with biliary and dietary cholesterol. It was shown that daily intake of 2–3 g of phytosterols decreases LDL cholesterol by 10–15% (31), while EFSA had authorized two health claims. The first points out “the beneficial effect of 0,8 g phytosterols and stanols on maintenance of normal blood cholesterol concentrations,” while the second emphasizes the “hypocholesterolemic effect of 1.5–2.4 g phytosterol daily intake” (28). Content of phytosterols in recommended daily portion (28 g) of nuts and seeds obtained in our study (Table 2) revealed that maximal intake of sterols would still be significantly lower (*ca*. 0,1 g) compared with amounts necessary to acquire the abovementioned beneficial effects. Also, though phytosterol content in nuts and seeds obtained in our study is substantially lower compared to those with proven beneficial physiological effects and compared with amounts obtained from plant oils, their positive effects on health in combination with other biologically active constituents from nuts and seeds should not be neglected.

According to the USDA Guideline for Americans, one ounce of nuts and seeds (about 28 g) can meet one-third of the adult protein needs for men and two-fifth for women (5, 6), based on data presented as a percentage of recommended intake (% RI) for proteins (Table 3) it is evident that this contribution from our samples is twofold lower. Analyzed samples have somewhat lower % EAAR in total proteins (Table 3) compared to some literature data for nuts (33), with the main limiting AA being methionine and lysine, while other authors (33, 34) report lysine as the main limiting AA in nut samples from the United States and Italy, respectively.

Still, besides protein content, an important parameter is the protein’s biological value, which is determined by EAAs content. The content of EAAs methionine is 2–4 fold lower in nut proteins compared to animal proteins, but this deficiency could be beneficial. Namely, available studies with restriction in methionine intake showed a decrease in mitochondrial production of reactive oxygen species and mitochondrial DNA mutations that at least partly could be responsible for the age-delaying effect observed in an animal model of methionine deficit diet (14). On the other hand, an animal model methionine fortified diet caused neurotoxic, including oxidative stress, inflammation, increased levels of β-amyloid peptide, and memory impairment (14, 35). Nuts and seeds are a rich source of non-essential AAs, especially arginine, asparagine, and glutamine (Supplementary Table 4). Arginine is nitric oxide (NO) precursor, which is a potent vasodilatory agent responsible for the regulation of vascular function and blood pressure (36), and for increased sensitivity in diabetic patients (37).

The low lysine/arginine ratio observed in nuts could have beneficial effects on preventing diabetes development (38). Furthermore, the lower lysine/arginine ratio in nuts and seeds’ proteins compared to animal proteins is linked to a significantly lower risk of hypercholesterolemia and atherosclerosis and, consequently, cardiovascular diseases (14). According to the obtained data (Supplementary Table 4), the lowest lysine/arginine ratio is in almonds, hazelnuts, walnut, and brazil nuts (0.20), while it is several times higher in pistachios (ca. 0.57) and cashew nut (0.45). In seeds, this ratio is ranged between 0.30 (roasted sunflower seed) and 0.56 (hemp seed).

Our study showed that nuts and seeds contain all amino acids necessary in the human diet and are unvarying considering total protein content, with somewhat higher values for EAAs in entire protein content within seeds (Supplementary Table 4). Differences among analyzed samples of nuts and seeds consider limiting AA, pointing out once again the importance of seeds within a well-balanced diet, first of all, the importance of their combining with nuts to provide adequate intake of all EAAs, especially within plant-based dietary regimes.

Mineral content in nuts and seeds per one recommended portion is expressed as a percentage of contribution to NRV (% NRV). These values are established to declare foodstuffs and as guidance level for consumers on daily intake of micronutrients for the average adult to prevent deficiencies (20), allowing the perceiving role of certain nuts and seeds in obtaining daily needs from minerals. Generally, nuts are considered one of the most important sources of magnesium in the diet; thus, the beneficial cardiometabolic effects of nuts are partially attributed to their magnesium content (39, 40). Several publications and meta-analyses showed an inverse association between magnesium intake and cardiovascular disease risk (41). Based on data from these investigations, it could be concluded that seeds are generally a more significant source of magnesium and, on average, contain twofold higher magnesium content than nuts.

Almonds, brazil nuts, and hazelnuts from our study were recognized as the richest sources of calcium, in accordance with some previous results of other authors (1, 29). The calcium content in almond samples from our study was twofold higher than the results mentioned earlier but in agreement with data from USDA Nutrient Database for Standard Reference (24). It could also be observed that seeds may be considered a more significant source of calcium than nuts (Table 4). It is also important to emphasize that in some nuts and seed samples (Supplementary Table 5) calcium content was higher than in dairy products, ranging from 120 to 800 mg/100 g, depending on the type of dairy product (42). It should be kept in mind that total mineral content is not the only parameter for defining some foodstuff as its’ rich source, but bioavailability should also be considered. That is the case with plant sources of calcium characterized by low bioavailability (42). Also, based on investigations performed by Siliburska and colleagues in which the bioaccessibility of minerals from different plant sources, including nuts, was assessed using *in vitro* enzyme digestion model, the highest bioaccessibility of calcium was reported in brazil nuts. It was substantially higher than other nuts analyzed (hazelnut, walnut, and cashew nut) (43). Moreda-Pineiro and colleagues, using *in vitro* dialyzability method to assess the bioavailability ratios of essential and toxic elements, showed that bioavailability and metal dialyzability varies among nuts and seed, which could be explained by their different chemical constituent, as well as by the negative influence of fat content (44). These authors showed that the highest bioavailability of calcium was from pistachios, while other examined minerals had better bioavailability from raw and roasted hazelnuts. On the contrary, for the majority of examined minerals, the lowest bioavailability was observed in walnut. This should be especially considered in individuals with intolerance/allergy to dairy products or on a vegan diet regime, whose only dietary sources of calcium are plant-based foodstuffs.

It was also shown that some representatives of nuts and seeds could provide up to one-fourth of daily needs for iron in one recommended portion (i.e., raw almonds and hemp seeds, 21.4 and 30.4% NRV, respectively), but in general, iron absorption is very low from plant food, that is when the iron is present in the non-hem form. Plant food, such as nuts and seeds, contain phytates, tannins, and fibers that significantly inhibit iron absorption, causing low bioavailability (44). In any case, regular consumption of nuts and seeds, especially almonds and pumpkin seeds, could contribute to the daily need for iron in persons who follow a vegan diet regime.

Nuts and seeds could be considered important sources of dietary zinc, which could be significant when planning dietary interventions for the population with zinc deficiencies. Results of previous studies conducted on the Serbian population showed that zinc is insufficiently present in the soil, water, and food and that serum zinc levels in the Serbian population are twofold lower compared to some other examined populations (45). Kafaoglu and colleagues, using *in vitro* intestinal method to establish bio element concentration dissolved in gastric and intestinal solution, have shown that the highest zinc bioavailability was obtained from hazelnuts and pistachios (46). All this implies that despite lower zinc content in nuts and seeds compared to other minerals, its content could be compensated by its higher bioavailability, and nuts and seeds could be recommended as an additional source of zinc in nutrition. Almost all analyzed samples could be considered significant copper and manganese sources (except boiled almonds and pistachios).

Observed differences in mineral content between raw and thermally treated samples of the same nuts and seeds could be explained in one part with the processing of the sample. For example, the lower content of some minerals in boiled peanuts is explained by the leaching of the mineral in water during the cooking process (47). Some data suggest that roasting could decrease the bioavailability of minerals since the structure of foodstuff and nutrients is changed so that digestive enzymes’ actions, and consequently bioaccessibility of minerals, are reduced (48).

Content of dietary fibers in commercial samples of nuts and seeds is mostly in agreement with United States Nutrient Database (24), except for chia seeds characterized by substantially higher fibers content (37.7 *vs.* 27.3 g/100 g) and pumpkin seeds with lower fibers content (3.9 *vs.* 6.5 g/100 g) (Supplementary Table 6). In general, nuts are considered one of the most important sources of dietary fibers, next to wholegrain cereals, but seeds seem to be more significant. Actual recommendations on fiber intake necessary for obtaining good health and recommended by various scientific authorities range from 25 to 35 g/day, that is between 10 and 13 g of fibers per 1,000 kcal (49). Accordingly, based on obtained results (Table 5), the fiber content in nuts and seeds could be considered significant and could increase dietary fiber intake. *In vitro* and *in vivo* studies showed that non-digestible components of nuts, such as dietary fibers, act as prebiotics that could modulate the gut microbiota profile, thus aiding intestinal homeostasis. Studies on walnut, pistachios, and almonds have shown that consumption over 3–16 weeks increases beneficial intestinal bacteria, especially butyric acid-producing strains. Moreover, intestinal microbiota-positive remodeling potential was found higher for almonds compared to commercially available fructooligosaccharides with previously proven prebiotic effects (3, 50).

In recent years, many authors have explained specific positive effects of nutrients by calculating the ratio of carbohydrates/fibers (51, 52). Fontanelli and colleagues have shown that increased consumption of grains, with a total carbohydrates/fibers ratio ≤ 10:1, influences cardiometabolic risk factors (atherogenic dyslipidemia and insulin resistance) by lowering blood triacylglycerols and fasting insulin (51). In our samples, this ratio was far below 10:1, while in chia, flax, and sesame seeds, it was almost 1:1. This is confirmed by their position in the PCA plot (Figure 4B). Our research is the first to represent carbohydrates/fibers and fibers/sugars ratios in nuts and seeds. Identical explanation of grains effects should be applied during monitoring of nuts and seeds effects considering calculations on carbohydrates/fibers and fibers/sugars ratio.

PCA established the results obtained in our study, and the conclusions were revealed. PCA confirmed nuts as the primary source of oleic acid and 11-eicosenoic acid (Figure 2A) and seeds as an important source of α-linolenic acid (Figure 2B). Also, PCA revealed that nuts and seeds are sources of different amino acids (Figures 1A,B), while some nuts and seeds samples are good sources of calcium and zinc.

## Conclusion

This research represents a comprehensive and comparative overview of the chemical composition and nutritional characteristics of nuts and seeds available on the Serbian market. Our results show that nuts and seeds are different, but also complementary in their composition. Based on our results, especially those related to the content of amino acids, it can be concluded that as far as satisfying the need for essential amino acids are concerned, it is beneficial to combine nuts and seeds due to their differences in essential amino acids. Moreover, our study highlights the importance of seeds in the diet, especially as a better source of omega-3 fatty acids, dietary fibers, calcium, magnesium, and zinc compared to nuts. Furthermore, based on our knowledge, this paper for the first time presents data related to the carbohydrates/fibers and fibers/sugars ratios in nuts and seeds, which additionally confirm their favorable nutritional characteristics.

## Data availability statement

The original contributions presented in this study are included in the article/Supplementary material, further inquiries can be directed to the corresponding author/s.

## Author contributions

NDjI, MD, and JKM: conceptualization, methodology, and writing—original draft preparation, review and editing. NDjI, MD, JKM, MJ, IS, and VT: formal analysis. BDj, NDjI, and MD: resources. BDj and VT: visualization. NDjI: supervision. All authors have read and agreed to the published version of the manuscript.
