# Supplementary material for: Similarities and differences in the nutritional composition of nuts and seeds in Serbia
Source: Front Nutr. 2022 Sep 16;9:1003125. doi: 10.3389/fnut.2022.1003125 (PMC9523690; doi:10.3389/fnut.2022.1003125)
Supplement: Supplementary file 1 [file Data_Sheet_1.pdf]

Table S1. Total fats and fatty acids content in analyzed samples (g 100 g^{-1})

Sample	Total fat	Saturated fatty acids*	Palmitoleic acid	Oleic acid	11-eicosenoic acid	Linoleic acid	Alpha-linolenic acid
Peanut raw	49.2±0.7	6.8±0.6	<0.05	23.8±0.7	0.66±0.06	15.6±0.9	<0.05
Peanuts roasted	52.5±0.6	8.7±0.5	<0.05	25.2±0.8	0.61±0.07	15.1±1.0	<0.05
Peanuts boiled	50.1±0.6	6.9±0.4	<0.05	23.9±0.6	0.60±0.07	15.8±0.8	<0.05
Almond raw	49.4±0.3	3.7±0.2	0.24±0.02	30.6±0.7	<0.05	12.1±0.6	<0.05
Almond roasted	55.2±0.3	4.2±0.3	0.22±0.03	34.6±0.5	<0.05	13.5±0.7	<0.05
Almond boiled	50.6±0.2	3.9±0.3	0.21±0.03	32.0±0.4	<0.05	12.1±0.5	<0.05
Hazelnuts raw	60.7±0.4	4.5±0.2	0.12±0.02	45.4±0.6	0.13±0.03	7.8±0.6	0.09±0.02
Hazelnuts roasted	62.4±0.8	4.5±0.4	0.12±0.02	46.4±0.5	0.13±0.03	8.4±0.4	0.06±0.02
Walnut	59.0±0.6	3.4±0.3	0.06±0.01	14.5±0.3	0.41±0.04	33.1±0.7	2.01±0.04
Brazil nut	66.4±0.4	15.1±0.4	0.23±0.03	24.2±0.4	0.05±0.02	20.5±0.4	<0.05
Cashew nut raw	43.8±0.9	7.8±0.4	0.14±0.02	23.5±0.3	0.14±0.03	7.8±0.6	0.06±0.02
Cashew nut roasted	47.8±0.7	8.5±0.3	0.15±0.02	25.6±0.3	0.15±0.03	8.5±0.5	0.07±0.02
Pecan nut	72.0±0.4	6.2±0.2	<0.05	40.6±0.4	0.21±0.03	20.6±0.9	0.99±0.04
Pine nuts	68.4±0.3	4.9±0.3	<0.05	17.9±0.4	0.80±0.04	33.1±1.0	0.11±0.03
Pistachios roasted	44.4±0.5	5.4±0.4	0.46±0.04	22.7±0.4	0.17±0.03	13.2±0.8	0.25±0.03
Sunflower seed raw	51.5±0.4	4.5±0.3	<0.05	18.4±0.3	0.09±0.02	23.0±0.8	0.07±0.02
Sunflower seed roasted	51.3±0.5	7.1±0.4	0.06±0.01	7.9±0.5	0.08±0.01	34.2±0.6	0.08±0.02
Pumpkin seed raw	45.8±0.8	8.7±0.3	0.10±0.01	14.1±0.4	0.10±0.02	20.7±0.8	0.18±0.03
Pumpkin seed roasted	42.1±0.6	8.0±0.4	0.09±0.02	13.0±0.4	0.10±0.01	19.0±0.7	0.17±0.02
Chia seed	30.8±0.7	3.2±0.2	<0.05	2.0±0.1	0.05±0.01	5.8±0.6	17.6±0.1
Flax seed	42.2±0.4	3.7±0.2	0.20±0.02	7.1±0.3	0.10±0.02	7.7±0.4	23.2±0.9
Hemp seed	48.8±0.5	4.6±0.3	0.30±0.03	5.3±0.2	0.12±0.02	27.5±0.7	8.7±0.8
Sesame seed	49.7±0.4	7.0±0.4	0.15±0.03	18.5±0.3	0.07±0.02	21.4±1.1	0.38±0.04
Black sesame seed	64.0±0.5	8.9±0.4	0.20±0.03	25.9±0.4	0.20±0.03	27.6±0.9	0.50±0.04
**Nuts/seeds****	**1.2**	**1.0**	**1.06**	**2.3**	**2.72**	**0.8**	**0.04**

*expressed as a sum of of myristic, palmitic, stearic and arachidic acid; **the ratio of the average value of specific nut parameters to the average value of specific seed parameters

Table S2. Phytosterols content in analyzed samples, reported as mg 100 g^{-1}

	Squalene	Campesterol	Stigmasterol	β-Sitosterol	Isofucosterol	Cycloartenol	Δ7-Avenasterol	Citrostadienol	Total sterols
Peanut raw	13.1	17.5	14.9	70.6	11.3	1.9	0.5	0.8	130.6
Peanuts roasted	8.4	15.5	13.9	65.9	9.6	1.5	0.5	0.8	116.1
Peanuts boiled	12.5	14.3	11.7	58.0	8.5	0.6	0.3	0.6	106.5
Almond raw	3.0	4.9	0.1	135.8	17.7	1.8	2.5	1.5	167.3
Almond roasted	3.5	4.7	1.2	144.1	16.6	3.6	3.3	2.8	179.8
Almond boiled	3.1	3.2	<0.3	112.3	11.3	3.1	2.9	2.4	138.3
Hazelnuts raw	15.8	5.7	1.5	105.0	3.0	<0.3	0.9	3.5	135.5
Hazelnuts roasted	21.6	5.7	18.9	85.4	2.1	<0.3	0.4	2.2	136.4
Walnut	<0.3	4.2	<0.3	84.4	7.6	9.0	<0.3	2.9	108.4
Brazil nut	111.4	<0.3	6.6	11.9	<0.3	0.3	0.4	2.3	133.1
Cashew nut raw	4.2	6.1	<0.3	77.2	8.7	3.7	<0.3	1.1	101.2
Cashew nut roasted	3.2	5.2	<0.3	60.0	7.1	2.9	<0.3	0.8	79.3
Pecan nut	7.2	4.1	2.4	99.9	12.2	2.4	0.4	5.0	133.6
Pine nuts	<0.3	16.6	<0.3	138.6	102.1	3.9	2.7	13.3	277.4
Pistachios roasted	5.6	13.2	2.5	206.1	17.8	<0.3	1.2	6.2	252.6
Sunflower seed raw	10.1	17.6	17.4	139.8	5.1	39.0	9.3	29.2	267.6
Sunflower seed roasted	7.8	18.5	15.2	140.7	9.8	26.5	8.5	19.4	246.4
Pumpkin seed raw	32.8	<0.3	<0.3	106.5	<0.3	<0.3	26.7	<0.3	166.3
Pumpkin seed roasted	31.1	<0.3	<0.3	98.6	<0.3	<0.3	25.5	<0.3	155.5
Chia seed	0.3	<0.3	<0.3	134.1	<0.3	<0.3	1.4	7.4	143.3
Flax seed	0.8	43.3	20.8	93.3	20.5	4..2	<0.3	3.0	222.9
Hemp seed	0.4	27.8	3.8	134.9	11.5	6.4	1.1	3.3	189.3
Sesame seed	1.0	75.6	28.7	304.6	56.0	4.9	5.8	12.9	489.6
Black sesame seed	0.9	84.2	34.1	287.7	79.2	4.8	4.5	10.0	505.5
LOQ	0.3	0.3	0.3	0.3	0.3	0.3	0.3	0.3	-

Nuts/seeds*	1.5	0.3	0.4	0.6	0.8	0.2	0.1	0.3	0.6

LOQ, limit of quantification; *the ratio of the average value of specific nut parameters to the average value of specific seed parameters

Table S3a. The essential amino acids content in analyzed samples, reported as g 100 g^{-1}

Samples	Lys	Thr	Val	Ile	Leu	Met	His	Phe
Peanut raw	1.08± 0.216	0.87± 0.174	1.07± 0.214	1.25± 0.250	1.97± 0.394	0.4± 0.08	0.71± 0.142	1.36± 0.272
Peanuts roasted	0.65± 0.130	0.75± 0.150	0.66± 0.132	2.42± 0.484	2.15± 0.430	0.75± 0.150	0.53± 0.106	1.04± 0.208
Peanuts boiled	1.04± 0.208	0.98± 0.196	1.05± 0.210	0.97± 0.194	1.7± 0.34	0.19± 0.038	0.81± 0.162	1.46± 0.292
Peanuts marinated	1.04±0.152	0.65± 0.110	0.96± 0.174	1.02± 0.182	1.65± 0.276	0.19± 0.028	0.71± 0.116	1.41± 0.236
Almond raw	0.76±0.14	0.55± 0.106	0.87± 0.164	0.91± 0.144	1.38± 0.298	0.14± 0.030	0.58± 0.136	1.18± 0.258
Almond roasted	0.70± 0.154	0.53± 0.182	0.82± 0.194	0.72± 0.162	1.49± 0.474	0.15± 0.066	0.68± 0.132	1.29± 0.256
Almond boiled	0.77± 0.106	0.91± 0.102	0.97± 0.146	0.81± 0.124	2.37± 0.222	0.33± 0.032	0.66± 0.10	1.28± 0.148
Hazelnuts raw	0.53± 0.112	0.51± 0.122	0.73± 0.150	0.62± 0.132	1.11± 0.238	0.16± 0.042	0.5± 0.110	0.74± 0.182
Hazelnuts roasted	0.56± 0.124	0.61± 0.124	0.75± 0.148	0.66± 0.136	1.19± 0.238	0.21± 0.04	0.55± 0.112	0.91± 0.170
Walnut	0.62± 0.082	0.62± 0.106	0.74± 0.094	0.68± 0.182	1.19± 0.228	0.2± 0.138	0.56± 0.074	0.85± 0.092
Brazil nut	0.41± 0.22	0.53± 0.148	0.47± 0.222	0.91± 0.264	1.14± 0.290	0.69± 0.054	0.37± 0.12	0.46± 0.194
Cashew nut raw	1.10± 0.212	0.74± 0.192	1.11± 0.194	1.32± 0.272	1.45± 0.336	0.27± 0.088	0.6± 0.108	0.97± 0.176
Cashew nut roasted	1.06± 0.070	0.96± 0.054	0.97± 0.072	1.36± 0.108	1.68± 0.132	0.44± 0.028	0.54± 0.066	0.88± 0.088
Pecan nut	0.35± 0.138	0.27± 0.112	0.36± 0.128	0.54± 0.130	0.66± 0.222	0.14± 0.06	0.33± 0.088	0.44± 0.112
Pine nuts	0.69± 0.226	0.56± 0.172	0.64± 0.2	0.65± 0.296	1.11± 0.352	0.3± 0.090	0.44± 0.10	0.56± 0.184
Pistachios roasted	1.13± 0.18	0.86± 0.244	1± 0.256	1.48± 0.218	1.76± 0.398	0.45± 0.144	0.5± 0.196	0.92± 0.270
Sunflower seed raw	0.94± 0.134	0.86± 0.144	0.99± 0.168	0.88± 0.244	1.33± 0.274	0.43± 0.090	0.8± 0.134	0.98± 0.166
Sunflower seed roasted	0.67± 0.230	0.72± 0.212	0.84± 0.28	1.22± 0.510	1.37± 0.64	0.45± 0.178	0.67± 0.188	0.83± 0.302
Pumpkin seed raw	1.15± 0.294	1.06± 0.246	1.4± 0.386	2.55± 0.330	3.2± 0.596	0.89± 0.152	0.94± 0.222	1.51± 0.416
Pumpkin seed roasted	1.47± 0.230	1.23± 0.212	1.93± 0.152	1.65± 0.122	2.98± 0.212	0.76± 0.10	1.11± 0.134	2.08± 0.188
Chia seed	1.15± 0.194	1.06± 0.174	0.76± 0.2	0.61± 0.224	1.06± 0.270	0.5± 0.088	0.67± 0.112	0.94± 0.182
Flax seed	0.97± 0.328	0.87± 0.224	1± 0.378	1.12± 0.298	1.35± 0.392	0.44± 0.202	0.56± 0.212	0.91± 0.406
Hemp seed	1.64± 0.168	1.12± 0.156	1.89± 0.204	1.49± 0.164	1.96± 0.226	1.01± 0.134	1.06± 0.122	2.03± 0.238

Sesame seed	0.90± 0.188	1.22± 0.172	1.28± 0.198	1.09± 0.176	1.99± 0.266	0.72± 0.086	0.98± 0.16	1.35± 0.196
Black sesame seed	0.84± 0.168	0.78± 0.214	1.02± 0.156	0.82± 0.152	1.13± 0.204	0.67± 0.224	0.61± 0.148	1.19± 0.164
Nuts/seeds*	0.71	0.69	0.66	0.80	0.82	0.49	0.68	0.73

Lys. lysin; Thr, threonin; Val, valine; Ile, isoleucine; Leu, leucine; Met, methionine; His, histidine; Phe, phenylalanine;
*the ratio of the average value of specific nut parameters to the average value of specific seed parameters.

Table S3b. The non-essential amino acids content in analyzed samples, reported as g 100 g^{-1}

Samples	Ser	Pro	Gly	Ala	Glu	Asp	Cys	Tyr	Arg
Peanut raw	1.68± 0.336	1.84± 0.368	1.91± 0.382	1.29± 0.258	5.48± 1.096	3.34± 0.668	0.45± 0.090	0.7± 0.14	3.08± 0.616
Peanuts roasted	1.06± 0.212	1.21± 0.242	1.49± 0.298	1.17± 0.234	6.25± 1.250	3.39± 0.678	0.27± 0.054	0.77± 0.154	2.64± 0.528
Peanuts boiled	1.28± 0.256	1.07± 0.214	2.00± 0.4	1.5± 0.30	6.02± 1.204	3.73± 0.746	0.35± 0.070	0.75± 0.150	3.76± 0.752
Peanuts marinated	1.21± 0.16	1.03± 0.162	1.53± 0.26	1.16± 0.210	6.33± 1.282	3.81± 0.518	0.29± 0.058	0.68± 0.088	3.42± 0.682
Almond raw	0.8± 0.198	0.81± 0.178	1.3± 0.302	1.05± 0.218	6.41± 1.464	2.59± 0.546	0.29± 0.066	0.44± 0.10	3.41± 0.702
Almond roasted	0.99± 0.170	0.89± 0.208	1.51± 0.378	1.09± 0.276	7.32± 1.148	2.73± 0.496	0.33± 0.06	0.5± 0.098	3.51± 0.762
Almond boiled	0.85± 0.142	1.04± 0.12	1.89± 0.144	1.38± 0.156	5.74± 0.666	2.48± 0.318	0.3± 0.058	0.49± 0.070	3.81± 0.536
Hazelnuts raw	0.71± 0.182	0.6± 0.138	0.72± 0.162	0.78± 0.178	3.33± 0.724	1.59± 0.336	0.29± 0.062	0.35± 0.066	2.68± 0.464
Hazelnuts roasted	0.91± 0.196	0.69± 0.142	0.81± 0.178	0.89± 0.166	3.62± 0.666	1.68± 0.346	0.31± 0.058	0.33± 0.090	2.32± 0.546
Walnut	0.98± 0.098	0.71± 0.096	0.89± 0.176	0.83± 0.190	3.33± 0.68	1.73± 0.330	0.29± 0.058	0.45± 0.062	2.73± 0.416
Brazil nut	0.49± 0.206	0.48± 0.154	0.88± 0.176	0.95± 0.18	3.4± 0.870	1.65± 0.358	0.29± 0.076	0.31± 0.078	2.08± 0.484
Cashew nut raw	1.03± 0.206	0.77± 0.170	0.88± 0.242	0.9± 0.250	4.35± 0.828	1.79± 0.368	0.38± 0.070	0.39± 0.076	2.42± 0.484
Cashew nut roasted	1.03± 0.082	0.85± 0.068	1.21± 0.084	1.25± 0.082	4.14± 0.610	1.84± 0.304	0.35± 0.026	0.38± 0.056	2.42± 0.274
Pecan nut	0.41± 0.154	0.34± 0.138	0.42± 0.146	0.41± 0.188	3.05± 0.730	1.52± 0.328	0.13± 0.054	0.28± 0.082	1.37± 0.436
Pine nuts	0.77± 0.238	0.69± 0.212	0.73± 0.244	0.94± 0.262	3.65± 0.930	1.64± 0.402	0.27± 0.052	0.41± 0.068	2.18± 0.4
Pistachios roasted	1.19± 0.248	1.06± 0.216	1.22± 0.210	1.31± 0.234	4.65± 1.064	2.01± 0.474	0.26± 0.130	0.34± 0.142	2± 0.506
Sunflower seed raw	0.86± 0.14	0.89± 0.148	1.25± 0.214	1.18± 0.190	4.24± 0.862	1.89± 0.384	0.39± 0.184	0.48± 0.082	2.06± 0.454
Sunflower seed roasted	0.7± 0.288	0.74± 0.286	1.07± 0.354	0.95± 0.324	4.31± 1.234	1.92± 0.610	0.92± 0.070	0.41± 0.152	2.27± 0.552
Pumpkin seed raw	1.44± 0.348	1.43± 0.286	1.77± 0.432	1.62± 0.358	6.17± 1.298	3.05± 0.670	0.35± 0.088	0.76± 0.198	2.76± 0.612
Pumpkin seed roasted	1.74± 0.242	1.43± 0.164	2.16± 0.170	1.79± 0.232	6.49± 0.826	3.35± 0.394	0.44± 0.062	0.99± 0.092	3.06± 0.428
Chia seed	1.21± 0.198	0.82± 0.168	0.85± 0.328	1.16± 0.262	4.13± 1.058	1.97± 0.490	0.31± 0.046	0.46± 0.08	2.14± 0.452

Flax seed	0.99± 0.44	0.84± 0.286	1.64± 0.22	1.31± 0.270	5.29± 1.362	2.45± 0.782	0.23± 0.112	0.4± 0.206	2.23± 0.588
Hemp seed	2.2± 0.224	1.43± 0.148	1.1± 0.152	1.35± 0.214	6.81± 0.832	3.91± 0.336	0.56± 0.08	1.03± 0.108	2.91± 0.490
Sesame seed	1.24± 0.172	1.08± 0.178	1.05± 0.250	1.17± 0.236	5.32± 0.848	2.37± 0.378	0.65± 0.078	0.71± 0.096	2.53± 0.412
Black sesame seed	1.12± 0.226	0.74± 0.134	0.76± 0.122	1.07± 0.238	4.16± 0.832	1.68± 0.336	0.4± 0.08	0.54± 0.108	2.45± 0.490
Nuts/Seeds*	0.74	0.83	0.92	0.81	0.90	0.90	0.64	0.72	1.08

Ser, serine, Pro, proline; Gly, glycine, Ala, alanine; Glu, glutamic acid; Asp, aspartic acid; Cys, cysteine; Tyr, tyrosine; Arg, arginine;
*the ratio of the average value of specific nut parameters to the average value of specific seed parameters;

Table S4. Total proteins and total amino acids content in analyzed samples, reported as g 100 g^{-1}

Samples	Total proteins	Total AAs	% EAAs	%NEAAs	EAAs/NEAAs*	%Basic AAs	% Aromatic AAs	% AcidicAAs	% Sulfur containing AAs	% Hydrophilic AAs	% Aliphatic AAs	Lys/Arg**
Peanut raw	29.23±0.16	28.48	30.58	69.42	0.44	17.10	7.23	8.82	2.98	11.46	32.76	0.35
Peanuts roasted	28.61±0.15	27.20	32.90	67.10	0.49	14.04	6.65	9.64	3.75	15.81	3346	0.25
Peanuts boiled	29.58±0.13	28.66	28.61	71.39	0.40	19.57	7.71	9.75	1.88	13.09	28.93	0.28
Almond raw	24.35±0.18	23.47	27.14	72.86	0.37	20.24	6.90	9.00	1.83	18.04	26.93	0.22
Almond roasted	25.95±0.22	25.25	25.27	74.73	0.34	19.37	7.09	10.05	1.90	17.07	25.82	0.20
Almond boiled	26.77±0.62	26.08	31.06	68.94	0.45	20.09	6.79	8.22	2.42	15.21	32.44	0.20
Hazelnuts raw	16.92±0.15	15.95	30.72	69.28	0.44	23.26	6.83	4.92	2.82	13.87	28.59	0.20
Hazelnuts roasted	18.12±0.15	17.00	32.00	68.00	0.47	20.18	7.29	5.30	3.06	11.92	29.35	0.24
Walnut	18.82±0.20	17.40	31.38	68.62	0.46	22.47	7.47	5.06	2.82	11.76	28.97	0.23
Brazil nut	16.28±0.24	15.51	32.11	67.89	0.47	18.44	4.96	5.05	6.32	15.96	31.14	0.20
Cashew nut raw	21.51±0.46	20.47	36.93	63.07	0.59	20.13	6.64	6.14	3.18	12.15	31.41	0.45
Cashew nut roasted	22.02±0.48	21.36	36.94	63.06	0.59	18.82	5.90	5.98	3.70	11.07	34.27	0.44
Pecan nut	11.69±0.39	11.02	28.04	71.96	0.39	18.60	6.53	4.57	2.45	17.19	24.77	0.26
Pine nuts	17.46±0.24	16.23	30.50	69.50	0.44	20.39	5.98	5.29	3.51	13.13	29.33	0.32
Pistachios roasted	23.02±0.37	22.14	36.59	63.41	0.58	16.40	5.69	6.66	3.21	11.23	35.37	0.57
Sunflower seed raw	21.12±0.29	20.45	35.26	64.74	0.54	18.58	7.14	6.13	4.01	12.28	31.88	0.46
Sunflower seed roasted	20.51±0.34	20.06	33.75	66.25	0.51	18.00	6.18	6.23	6.83	14.44	30.86	0.30
Pumpkin seed raw	33.29±±0.43	32.05	39.63	60.37	0.66	15.13	7.08	9.22	3.87	13.32	37.35	0.42
Pumpkin seed roasted	35.82±0.28	34.66	38.11	61.89	0.62	16.27	8.86	9.84	3.46	12.06	34.45	0.48

Chia seed	20.52±0.31	19.80	34.09	65.91	0.52	20.00	7.07	6.10	4.09	9.04	26.57	0.54
Flax seed	23.59±0.36	22.60	31.95	68.05	0.47	16.64	5.80	7.74	2.96	12.68	32.12	0.43
Hemp seed	34.23±0.23	33.50	36.42	63.58	0.57	16.75	9.13	10.72	4.69	10.31	27.52	0.56
Sesame seed	26.61±0.43	25.65	37.15	62.85	0.59	17.19	8.03	7.69	5.34	10.82	29.86	0.36
Black sesame seed	20.76±0.35	19.98	35.34	64.66	0.55	19.52	8.66	5.84	5.36	10.93	27.73	0.34

AAs, amino acids; EAAs, essential amino acids; NEAAs, non-essential amino acids; *the ratio of essential amino acids to non-essential amino acids ; ** the ratio of lysine to arginine

Table S5. Macro and micro essential elements concentration in analyzed samples, reported as mg kg^{-1}

Sample	Na	K	Ca	Mg	P	Fe	Cu	Zn	Cr	Mo	Mn
Peanut raw	45.6	3508	647	1706	4221	35.6	10.4	34.5	0.44	0.88	13.7
Peanuts roasted	83.5	3771	726	1847	4587	37.9	9.9	36.7	0.37	1.93	14.4
Peanuts boiled	<25	3665	357	1556	3824	18.8	3.5	30.3	0.34	1.77	13.8
Almond raw	8.4	3937	1995	2655	5191	107.0	10.7	31.6	2.17	0.25	20.6
Almond roasted	21.1	4040	2301	2665	5324	79.2	9.6	32.3	1.89	0.46	20.7
Almond boiled	10.5	3995	2251	2660	5280	85.0	10.1	32.0	1.99	0.29	20.6
Hazelnuts raw	<25	3812	1388	1467	3412	46.2	13.3	23.1	1.70	<0.06	64.2
Hazelnuts roasted	<25	4024	1389	1514	3623	28.7	13.8	23.6	<0.25	<0.06	72.0
Walnut	<25	3997	974	1867	5368	35.1	15.8	36.4	1.01	<0.06	30.6
Brazil nut	<25	3760	1603	3724	7244	25.9	18.8	41.9	<0.25	<0.06	13.2
Cashew nut raw	61.7	3807	376	2374	5261	51.4	19.8	51.5	<0.25	<0.06	18.0
Cashew nut roasted	4412	3937	504	2501	5534	57.7	22.1	57.6	<0.25	<0.06	20.3
Pecan nut	<25	2295	532	1042	2814	26.0	7.5	37.6	<0.25	<0.06	35.5
Pine nuts	<25	3407	84.2	1936	4938	42.2	9.2	63.0	<0.25	<0.06	73.0
Pistachios roasted	800	4414	989	981	4590	26.9	4.6	21.4	0.31	<0.06	7.9
Sunflower seed raw	<25	3858	995	2813	7092	40.6	17.4	53.0	<0.25	0.38	21.3
Sunflower seed roasted	2550	2708	552	1839	4558	24.7	10.0	34.6	<0.25	0.26	15.1
Pumpkin seed raw	91.3	4083	574	4253	9290	70.0	9.0	33.1	<0.25	1.46	29.3
Pumpkin seed roasted	3310	4363	600	4344	9981	73.9	10.2	45.3	0.30	1.52	32.1
Chia seed	<25	4478	5836	3409	9234	62.1	14.2	52.5	0.32	0.58	41.2

Flax seed	709	4366	1576	3042	6191	55.0	10.6	50.5	0.27	<0.06	26.2
Hemp seed	10	4365	1400	5125	9685	152.0	12.6	76.5	0.48	0.74	60.2
Sesame seed	548	2927	630	2937	6475	56.3	15.4	55.5	<0.25	0.81	13.0
Black sesame seed	<25	2808	9247	2487	5484	55.7	13.2	41.1	<0.25	2.55	17.1
LOQ	25	10	25	10	2.75	0.5	0.5	0.05	0.25	0.06	0.05
Nuts/seeds*	0.5	1.0	0.5	0.6	0.6	0.7	1.0	0.8	3.1	0.4	1.0

LOQ, limit of quantification; *the ratio of the average value of specific nut parameters to the average value of specific seed parameters

Table S6. Total carbohydrates, fibers, sugars, sucrose, glucose, and fructose content in analyzed samples, reported as g 100 g^{-1}

Sample	Total carbohydrates	Total fibers	Total sugar*	Sucrose	Glucose	Fructose
Peanut raw	14.0±1.1	8.5±0.3	4.0±0.2	3.8±0.2	0.10±0.03	0.10±0.04
Peanuts roasted	16.3±0.8	8.4±0.2	4.2±0.2	4.0±0.1	0.10±0.03	0.10±0.02
Peanuts boiled	17.2±0.8	8.7±0.3	4.1±0.3	3.9±0.2	0.10±0.02	0.11±0.03
Almond raw	20.3±0.6	12.2±0.3	3.9±0.3	3.7±0.3	0.10±0.02	0.10±0.03
Almond roasted	15.4±0.6	10.5±0.3	4.4±0.3	4.3±0.2	<0.1	<0.1
Almond boiled	16.7±0.9	10.4±0.3	4.9±0.2	4.8±0.1	<0.1	<0.1
Hazelnuts raw	16.1±0.7	9.7±0.2	4.3±0.2	4.2±0.2	0.07±0.02	0.07±0.02
Hazelnuts roasted	15.9±1.1	9.4±0.3	4.9±0.1	4.8±0.1	0.07±0.03	0.07±0.03
Walnut	9.7±0.5	7.1±0.2	1.4±0.1	1.3±0.1	0.05±0.02	0.05±0.02
Brazil nut	12.1±0.8	7.5±0.4	2.3±0.1	2.3±0.1	<0.1	<0.1
Cashew nut raw	28.3±1.6	3.3±0.3	5.9±0.1	5.8±0.1	0.05±0.03	0.05±0.02
Cashew nut roasted	26.2±1.4	3.2±0.3	5.0±0.2	4.8±0.2	0.08±0.02	0.08±0.02
Pecan nut	13.9±0.9	9.6±0.6	4.0±0.3	3.9±0.3	<0.1	<0.1
Pine nuts	10.8±0.7	3.7±0.3	3.6±0.1	3.5±0.1	0.06±0.02	0.06±0.02
Pistachios roasted	27.4±1.0	10.3±0.3	7.6±0.3	7.0±0.2	0.31±0.03	0.21±0.07
Sunflower seed raw	21.2±0.7	8.6±0.4	2.6±0.1	2.5±0.1	<0.1	<0.1
Sunflower seed roasted	25.5±1.0	10.6±0.5	3.1±0.2	2.9±0.2	0.10±0.02	0.10±0.03
Pumpkin seed raw	12.2±1.6	3.9±0.4	1.9±0.3	1.0±0.1	0.40±0.10	0.50±0.09

Pumpkin seed roasted	12.7±1.1	3.9±0.4	2.1±0.3	1.4±0.2	0.31±0.08	0.40±0.04
Chia seed	41.5±1.3	37.7±1.4	<0.1	<0.1	<0.1	<0.1
Flax seed	27.0±1.2	25.2±0.8	1.7±0.2	1.2±0.1	0.40±0.06	0.10±0.03
Hemp seed	6.8±1.1	4.0±0.4	1.5±0.2	0.9±0.1	0.23±0.06	0.32±0.08
Sesame seed	17.6±0.9	11.8±0.5	0.3±0.1	0.2±0.1	0.05±0.02	0.05±0.03
Black sesame seed	10.5±1.2	10.0±0.4	0.20±0.04	0.10±0.02	0.05±0.03	0.05±0.02

* expressed as the sum of glucose, fructose and sucrose
